# Nutritional and Functional Properties of *Terminalia ferdinandiana* Fruits Wild Harvested from Western Australia

**DOI:** 10.3390/foods13182888

**Published:** 2024-09-12

**Authors:** Eshetu M. Bobasa, Anh Dao Thi Phan, Michael E. Netzel, Saleha Akter, Daniel Cozzolino, Yasmina Sultanbawa

**Affiliations:** ARC Industrial Transformation Training Centre for Uniquely Australian Foods, Centre for Nutrition and Food Sciences, Queensland Alliance for Agriculture and Food Innovation, The University of Queensland, Indooroopilly, QLD 4068, Australia; a.phan1@uq.edu.au (A.D.T.P.); m.netzel@uq.edu.au (M.E.N.); saleha.akter@uq.edu.au (S.A.); d.cozzolino@uq.edu.au (D.C.)

**Keywords:** *Terminalia ferdinandiana*, vitamin C, ellagic acid, sugar, functional properties

## Abstract

This study assessed the metabolite content and bioactivities of Kakadu plum (KP) from Western Australia (WA). LC-MS/MS and UHPLC-PDA analyzed sugar, vitamin C, and ellagic acid (EA). Functional properties were evaluated by spectroscopic technique, agar well diffusion, and microplate dilution methods. WA KP exhibited higher total sugar (16.3 ± 1.0 g/100 g DW) and free ellagic acid (EA) (23.2 ± 1.7 mg/g DW), along with abundant vitamin C (25.20 ± 0.16 to 131.50 ± 0.20 mg/g DW) compared to Northern Territory KP fruits. The fruit showed strong antioxidant activities, α-glucosidase inhibition, and effectiveness against bacteria, with positive correlations to total phenolic content (TPC), vitamin C, and EA. These findings highlight WA KP’s potential for functional foods and pharmaceuticals, emphasizing the importance of TPC, vitamin C, and EA in selecting high-quality fruit.

## 1. Introduction

Over the last decades, consumers have been increasingly interested in fruits and vegetables because of their potential to reduce the risks of chronic diseases [1]. This health advantage was linked to the presence of a broad range of metabolites such as carotenoids, fiber, lipid- and water-soluble vitamins, minerals, and polyphenols [2,3].

The concentration of metabolites in wild grown fruits and vegetables can vary considerably [4]. Saline soil [5], growing temperature, and ripening can cause increased phenolic acid, flavanol, and anthocyanin content, as well as overall antioxidant capacity [6]. It was also shown that environmental change affects metabolite gene expression [7]. Therefore, assessing the effects of region (geographical location) is important in terms of selecting the “best quality” fruit for functional food and/nutraceutical industries.

A growing amount of evidence has shown that *Terminalia ferdinandiana* (family *Combretaceae*; genus *Terminalia*), commonly known as Kakadu plum (KP), fruits are a uniquely highly edible source of vitamin C and are rich in EA (EA) and ellagitannins (ETs) [8]. Konczak et al. [4] also presented KP as a unique edible source of sugars. However, a great degree of variability exists among KP studies [4,9] that could have a significant impact on selecting quality fruit based on composition as well as functional properties. Our previous work [10] targeted fruits wild harvested from Northern Territory, where superior ETs, EA, and vitamin C composition and functional quality were reported. The present study aimed to evaluate the vitamin C, ellagic acid, and sugar composition (namely glucose, fructose, and sucrose) and functional properties of KP fruits wild harvested from Western Australia.

## 2. Materials and Methods

### 2.1. Plant Materials, Chemicals, and Reagents

Fully ripe and mature fruits of Kakadu plum (KP) were wild harvested from Western Australia (WA) and Northern Territory (NT). The Western Australian fruits were sourced from trees grown in two distinct locations: the Karajarri (K) country and the Yawuru (Y) conservation area within the Kimberley region. Meanwhile, fruits from the Northern Territory were obtained from Darwin. Ten KP trees were randomly chosen from each site, yielding 50–100 fruits per tree with yield of 150–290 g fruits. The harvested samples were transported to the laboratory while being kept cool, stored at −80 °C. After manually deseeding each fresh fruit, all the pulps (including peels) of each tree as composite were blended into a puree using mortar and pestle first, and then a Waring 8010S Laboratory Blender (Waring^®^ Laboratory Science, Torrington, CT, USA). The puree was then freeze-dried under vacuum (Lindner & May Ltd., Windsor, Brisbane, Australia) and subsequently processed into fine powder for analysis of sugar, vitamin C, and EA and functional properties.

Analytical-grade standards (purity > 99%) (glucose, sucrose, fructose, tannic acid, ellagic acid, gallic acid, L-ascorbic acid, ferrous sulphate) and chemicals including sulfuric acid (99.9%) were supplied by Sigma Aldrich (Castle Hill, New South Wales, Australia). Acarbose was from Bayer Pharmaceuticals, Leverkusen, Germany. HPLC-grade organic solvents (methanol, acetonitrile), formic acid, and reagents (Folin-Ciocalteu’s phenol and 2, 2-diphenyl-1-picrylhydrazyl (DPPH)) were also sourced from Sigma Aldrich. Sodium carbonate anhydrous were supplied by Chem-supply (Bedford St, Gillman, South Australia, Australia), and Trolox was obtained from Merck KGaA, Darmstadt, Germany.

Standard plate count agar (PCA-CM0463), Mueller-Hinton agar (MHA), and tryptone soya yeast extract broth (TSYEB) (CM 129B) were obtained from Oxoid Ltd. (Basingstoke, UK). The bacterial strains were from the American Type Culture Collection (ATCC; Oxoid Ltd., Basingstoke, UK) and the National Collection of Type Cultures (NCTC; Health Protection Agency Centre for Infection, London, UK), and clinical isolate was from the Royal Brisbane and Women’s Hospital (Herston, Queensland, Australia). They included Gram-positive strains *Staphylococcus aureus* (NCTC 6571), Methicillin resistant *Staphylococcus aureus* (MRSA) clinical isolates (CIII) (MRSA3) (18 October 2012), and *Bacillus cereus* (ATCC 10876), and Gram-negative strains *Acinetobacter baumannii* (ATCC 686), *Pseudomonas aeruginosa* clinical isolates (CI) (ATCC 9001), *Escherichia coli* (NCTC 9001), and *Shewanella putrefaciens* (ATCC 49138).

### 2.2. Total Water-Soluble Carbohydrate Content

Aqueous extracts were utilized to analyze total carbohydrate contents. Approximately 100 mg of freeze-dried WA fruit powder was extracted with 5 mL MilliQ water (Millipore, North Ryde, New South Wales, Australia) and analyzed following published procedures. Briefly, equal amounts of diluted extracts and 80% phenol were mixed. Then, 500 µL of concentrated H_2_SO_4_ was added, and the mixture was vortexed. The solution was left to cool for 30 min. The absorbance was monitored at 490 nm using a spectrophotometer (Tecan, Grödig, Austria) with a 96-well plate. Results are expressed as mg Glu E/g DW [11,12].

### 2.3. LC-MS/MS Analysis of Sugars

Accurately weighed samples (~0.1 g) were extracted with 10 mL of 70% methanol. The homogenate was thoroughly vortexed, placed in a reciprocating shaker (RP1812, Paton Scientific, Adelaide, SA, Australia) for 30 min, and then incubated for 15 min in a sonication bath (55 °C). The mixture was centrifuged (5000 rpm for 10 min) (Eppendorf Centrifuge 5804, Hamburg, Germany), and the extraction was repeated twice (10- and 5-mL solvents, respectively) following the same procedure. Finally, the combined supernatants were filtered through 0.2 µm PTFE filters (Millipore, New York, NY, USA) into HPLC vials and stored at −35 °C until analysis.

Individual sugars (glucose, fructose, and sucrose) were identified and quantified using a Shimadzu Nexera X2 UHPLC system coupled with a Shimadzu MS-triple quadrupole mass spectrometer (Shimadzu, Kyoto, Japan, System 3) with Lab Solutions software based on procedures described by Hong and colleagues [13] without modification. Two mobile phases were used for separation on an Acquity UPLC BEH Amide column (100 × 2.1 mm i.d., 1.7 µm particle size; Waters, Dublin, Ireland): Mobile Phase A consisted of 80% aqueous acetonitrile with 0.1% NH_4_OH, and Mobile Phase B was 0.1% NH_4_OH in MilliQ water. The elution was carried out at a flow rate of 0.2 mL/min. The column temperature was maintained at 40 °C.

Electrospray ionization (ESI) in negative ion mode was used, with a nebulizer gas flow rate of 3 L/min and a drying gas flow rate of 10 L/min. The desolvation line (DL) temperature was set at 250 °C, and the heat block temperature was 400 °C. The presence and abundance of the selected sugars were calculated by comparison of the peak area with the peak area of known standards using Chromeleon version 4.6 software. Fructose (2.164–216.4 µg/mL), glucose (2.052–205.2 µg/mL), and sucrose (2.604–260.4 µg/mL) standards were prepared in 50/50 acetonitrile/H2O (*v*/*v*) [14,15].

### 2.4. Analysis of Vitamin C and Ellagic Acid (EA) Content

The analyses were carried out as previously mentioned in Bobasa et al. [10] on each KP tree fruit sample. Briefly, 0.1 g of finely ground powder (vitamin C extraction) was mixed with 10 mL 3% MPA-8% Acetic Acid in 1 mM EDTA, vortexed (30 s), sonicated for 15 min, and centrifuged (4000 rpm, 15 min, 4 °C). The supernatant was collected, and the process repeated three times. The collected supernatant was subjected to reduction using 40 mM DL-Dithiothreitol (DTT; threo-1,4-Dimercapto-2,3-butanediol) (DTT) in Trizma buffer to obtain total vitamin C, whereas the powder (0.1 g) was mixed with AAE (80% *v*/*v* ethanol, 20% *v*/*v* water, 0.2% HCl), vortexed, sonicated (15 min), and centrifuged (4000 rpm, 15 min, 20 °C). Clear supernatants of the triplicate extraction were combined and used for EA analysis.

WatersTM UHPLC-PDA system (Waters, Milford, MA, USA) with Waters HSS-T3 column (150 × 2.1 mm i.d; 1.8 μm) maintained at 25 °C and Waters BEH Shield RP C18 column (100 × 2.1 mm i.d; 1.7 µm) maintained at 35 °C were used to identify and quantify vitamin C and ellagic acid, respectively, according to Bobasa et al. [10]. For vitamin C analysis, an isocratic mobile phase composed of aqueous 0.1% (*v*/*v*) formic acid at a flow rate of 250 μL/min was used during the separation. The injection volume was 2 μL, and vitamin C was quantified using an external calibration curve of ascorbic acid acquired at 245 nm, whereas ellagic acid was analyzed using 0.1% formic acid in Milli-Q water (mobile phase A) and 0.1% formic acid in methanol (mobile phase B). The flow rate was 0.3 mL/min with gradient elution for mobile phase B as follows: 35% B isocratic elution for 5 min, 50% B over 10 min, and 100% B by 15 min. The column was re-equilibrated for 7 min before the next injection. An aliquot of 2 μL of a sample was injected, and the chromatogram was extracted at 254 nm. An external calibration curve of EA was prepared to quantify the levels of ellagic acid.

### 2.5. Extract Preparation for Phenolic Content and Functional Properties

The extract was prepared in AAE (80% *v*/*v* ethanol, 20% *v*/*v* water, 0.2% HCl) following the procedure previously mentioned in Bobasa et al. [10]. These extracts were used for the experiments described under Section 2.6, Section 2.7, Section 2.8 and Section 2.9.

### 2.6. (Poly) Phenolic Content

#### 2.6.1. Determination of Total Phenolic Content (TPC)

The TPC was determined as previously described [10]. Appropriately diluted extracts (20 µL) were allowed to react with 125 µL 10% Folin–Ciocalteu (F-C) reagent and 125 µL 0.7 M Na_2_CO_3_ in the dark for 15 min. The absorbance was monitored at 700 nm against gallic acid standard (0–120 μg/mL prepared in milli-Q water), and results were expressed as gallic acid equivalent (GAE) per gram dry weight.

#### 2.6.2. Determination of Total Flavonoid Content (TFC)

The total flavonoid content (TFC) was ascertained using the classical colorimetric method [16]. Briefly, 100 μL of 2% aluminium trichloride (AlCl_3_) in methanol was mixed with 100 μL of the sample extracts. Absorption readings at 415 nm were taken after 10 min against a blank sample extract. Standard quercetin (10–200 mg/L in methanol) was used to report the results as mg quercetin equivalent (QE)/g DW [16].

### 2.7. Evaluation of Antioxidant Activities

#### 2.7.1. FRAP (Ferric Reducing Antioxidant Power of Plasma) Assay

The assay was conducted according to Benzie and Strain [17] with minor modifications. Water (30 µL), appropriately diluted fruit extracts (20 µL), and FRAP reagent (200 µL) were mixed in 96-well plates. Absorbance at 593 nm was measured after 6 min incubation using a microplate reader. Results are presented against FeSO_4_.7H_2_O standard curve (0.1 to 1 mM) as micromoles of Fe^2+^ equivalent per gram dry weight (µmole Fe^2+^ E/g DW).

#### 2.7.2. DPPH Radical Scavenging Power

DPPH radical scavenging assay was carried out using the Moore and Yu method as mentioned in our previous publication [10]. The extracting solvent was evaporated, and the sample reconstituted in methanol. The diluted extract radical scavenging capacity was measured at 517 nm after mixing with DPPH free radical. The results are expressed as Trolox equivalent (TE) per gram dry weight from Trolox standard curve (5 to 35 μmole/L).

### 2.8. Determination of Antimicrobial Activity

The extracts were freeze-dried and freshly reconstituted in 20% ethanol to give 40 mg/mL before antimicrobial analysis. The antimicrobial test was performed according to Bobasa et al. [10] without modification. In summary, the study used both Gram-positive and Gram-negative bacteria to test antimicrobial activity through a well diffusion assay. Bacterial strains were inoculated on agar plates and pre-cultured at appropriate temperature for 16 hrs. Each bacterial strain was prepared to a concentration of 10^4^ CFU/mL with sterile phosphate buffered saline (PBS) (0.9%) at 600 nm using a spectrophotometer (Genesys 20, Thermo Fisher Scientific Australia Pty Ltd., Melbourne, VIC, Australia). The bacterial suspensions were streaked onto MHA plates, and wells were punched into the agar. Extracts and standards were added to the wells, and the plates were incubated for 16 h at specific temperatures. The inhibition zones around the wells were measured to assess antimicrobial activity.

The minimum inhibitory concentration (MIC) was determined for the most sensitive strains using a microplate dilution method. Cultures of *S. aureus*, *S. putrefaciens*, and MRSA3 were incubated, and the MIC was identified as the lowest concentration with no visible bacterial growth.

### 2.9. In Vitro α-Glucosidase Inhibition Assay

The assay was carried out on 96-well microplates in accordance with the method described by Zhang et al. [18] using p-nitrophenyl-α-D-glucopyranoside (pNPG) as a substrate with slight modification. Briefly, α-glucosidase (α-glucosidase-EC 3.2.1.20) (30 μL, 1.0 unit/mL in 0.1 M phosphate buffer (pH 6.9)) was mixed with different concentrations of extracts (100 μL) (final well concentrations would be 375, 312.5, 156.3, 62.5, 31.3, 15.6, and 7.8 µg/mL) or acarbose (Bayer Pharmaceuticals, Leverkusen, Germany) in a 96-well plate for 10 min at 37 °C. After incubation, 2 mM pNPG (30 μL) was added, and the release of p-nitrophenol from pNPG was monitored at 405 nm after 20 min with a microplate reader against blank. The graph prism was used to calculate the IC_50_ of enzyme activity by converting the concentration to logarithm 10 vs. normalized response.

### 2.10. Statistical Analysis

Results are mean ± standard error of the mean (SEM) of triplicate measurements. Differences between means were determined by one-way analysis of variance (ANOVA) followed by Tukey post hoc test using IBM SPSS Statistics 25 (SPSS Inc. Chicago, IL, USA). Further correlations were obtained by Pearson correlation coefficient in bivariate correlations. Unscrambler software (version 11; CAMO Analytics, Oslo, Norway) and GraphPad Prism version 8 (La Jolla, CA, USA) were used to perform the PCA and IC50. A *p* < 0.05 was considered as significant.

## 3. Results and Discussion

### 3.1. Total Water-Soluble Carbohydrate and Sugar Contents

The results in Table 1 showed that the soluble carbohydrate in the two-sub location (≈38 g GluE/100 g DW), that is, 6% of fresh weight of edible flesh, is similar to the findings on cashews (38.61 g GluE/100 g DW) and tomatoes (42.36 g GluE/100 g DW) [19]. *Lycium barbarum* L. and *Lycium chinense* Mill. fruits, widely consumed by Chinese populations, have total carbohydrate contents in the range of 33–49 g GluE/100 g DW [20], in line with the carbohydrate content obtained in the present study (Table 1).

The glucose (66 mg/g DW) and fructose (73 mg/g DW) content recorded in WA fruit are two- and six-fold higher than that present in *T. chebula* fruit (glucose = 26 mg/g DW, fructose = 12 mg/g DW), respectively [21]. The sucrose content is 0.5 ± 0.03 g/100 g FW (Table 1), consistent with that reported in currant (0.60 ± 0.042 g/100 g FW) and wild green fig (0.50 ± 0.03 g/100 g FW) [22].

Fruits like tomatoes provide 2.8% fresh weight total soluble sugar [23] and are healthy sources of carbohydrates for diabetes patients because of low glycemic index (38.38 ± 1.42) [19]. Several studies have associated low glycemic index with high antioxidant content, including polyphenolic compounds and vitamin C, as well as abundance of soluble sugars [19]. These are well-known characteristics of KP (shown in Table 2 and Table 3), making it a suitable and healthy source of sugars for patients with diabetes. Consequently, KP has the potential to be expanded in its applications as a functional food.

Moreover, we found a notable difference in sugar content between the regions. WA fruits proved to be a richer source of sugar (16.3 ± 1.0 g/100 g DW) compared to those harvested in NT (5.2 ± 0.8 g/100 g DW). Zhang et al. [24] demonstrated that factors like longer sunshine hours, dry soil, and lower annual precipitation impacted blueberry sugar content. These same factors could influence the sugar variations observed across the two regions.

### 3.2. Vitamin C and Ellagic Acid Content

The results are illustrated in Table 2. For the ten trees analyzed for each location, the Yawuru sample gave total vitamin C (TVC) in the range of 42.5 ± 0.27 to 122.33 ± 3.25 mg/g DW (8.00 ± 0.05 to 19.30 ± 0.51 mg/g FW), while the contents in Karajarri fruits were from 25.20 ± 0.16 to 131.50 ± 0.20 mg/g DW (3.80 ± 0.02 to 19.73 ± 0.03 mg/g FW).

These findings are closer to the TVC reported by Williams et al. (140.38 ± 7.01 mg/g DW) [8]. Konzack et al. [4] found 159.1 ± 46.6 mg/g DW L-AA among fruits picked in the Broome peninsula, slightly higher than our report of the maximum yield shown in Table 2 (118.63 ± 2.75 mg/g DW, Yawuru; 110.70 ± 1.23, 111.51 ± 0.42, 126.43 ± 0.51 mg/g DW, Karajarri). However, our results in Table 2 show significantly higher vitamin C levels compared to those reported in common vegetables and fruits [25,26].

Apart from vitamin C, EA stands as a crucial non-nutrient plant metabolite. While the physiological importance of EA as a functional food ingredient is evident in its unbound state, a substantial quantity also exists either bound or in the form of ellagitannins [27]. The free form of EA (FEA) is dominant in WA KP fruit, accounting for 47–85% of the total ellagic acid (TEA), as shown in Table 2. These ratios are comparable with Williams and colleagues (FEA/TEA = 71%, KP fruit) [8], and that of blackberries, red raspberries, marionberries, and black raspberries (40–50% FEA/TEA) [28].

Despite the fact that our findings present Kimberly KP fruit as an outstanding source of free ellagic acid (FEA), notably, KP fruits, irrespective of their origin, stand out as a unique edible source of EA when compared to cloudberry, raspberry, strawberry, and sea buckthorn, which are typically consumed in Finland, exhibiting a range of 0.7 to 4.7 mg/100 g FW FEA [29].

A report on contents of TEA in fruits and nuts [27] indicated TEA as low as 1 mg/100 g FW for sea buckthorn and as high as 360 mg/100 g FW for cloudberry. Our results in Table 2 present the maximum TEA (Table 2) of 790 mg/100 g FW (Karajarri) and 833 mg/100 g FW (Yawuru), over two-fold higher than in cloudberries, which could further substantiate the Combretaceae family as a well-known source of EA and ETs [30].

### 3.3. Total Phenolic and Flavonoid Content

There is a global trend of increased fruit consumption to meet the daily phenolic requirements of 900 mg/day as a preventive measure against the onset of chronic disorders [31]. Multiple studies have been undertaken to measure the phenolic yield of fruits. Table 3 summarizes the TPC and TFC of KP fruits. The TPC were within the range of 78–195 mg GAE/g DW (12 to 31 mg GAE/g FW), which closely aligns with the TPC documented by Konczak et al. [4] (ranging from 132.53 ± 15.8 to 187.68 ± 30.8 mg GAE/g DW). Furthermore, Akter et al. [32] reported TPC of 120 mg GAE/g DW in KP fruits harvested in NT, a level comparable to our results.

Our findings, however, revealed the exceptional quality of KP fruits, characterized by elevated TPC levels as evidenced by its superior TPC (Table 3) relative to those found in cereals such as barley, buckwheat, wheat, and rye, ranging from 13.2 to 50.7 mg GAE/g DW [33]. Additionally, our values are 3 to 207 times higher than the TPC levels documented in various vegetables [34] and fresh fruits available in the Spanish market [35]. However, low levels of flavonoid contents 3 to 7 mg QE/g DW (42 to 118 mg QE/100 g FW) were found (Table 3).

### 3.4. Antioxidant Activities

Antioxidants prevent chronic diseases by interacting with free radicals. This is crucial, as excessive free radicals can damage DNA, proteins, and cell membranes, posing a significant health risk [36]. The results of the total reducing capacity (TRC) and DPPH radical scavenging are displayed in Table 3. The current minimum Karajarri tree fruit TRC value is 5- to 11-fold higher than riberry, finger lime (pink), finger lime (green), Davidson’s plum, and lemon aspen TRC [37]. Netzel et al. [38] reported TRC values in muntries, Tasmanian pepper, Illawarra plum, Cedar Bay cherry, and Burdekin plum in agreement with our results (Table 3). On the other hand, our results in Table 3 displayed superior reducing capacity compared to 52 fruits marketed in Spain, 0.08 to 86.34 μmole Fe^2+^/g FW [35], which makes KP a primary fruit to make significant contributions to functional food and/nutraceutical industries.

A Pearson correlation analysis (Table S1) showed a strong positive relation between FRAP and TPC and vitamin C contents (r > 0.90, *p* = 0.01), demonstrating phenolic compounds and vitamin C could be main contributors. A previous study also demonstrated that TPC and vitamin C found in fruits and vegetables positively influenced FRAP results (r = 0.95 to 0.99, *p* < 0.0001) [39].

Furthermore, Pfundstein et al. [40] indicated that ETs in Terminalia fruit exhibited the highest antioxidant activities because of the number of hydroxyl groups per molecule. Similarly, the DPPH radical scavenging potential of KP fruits was shown to be strongly (r = 0.82 and 0.73, *p* < 0.01) related to the TEA contents, obtained after ET acid hydrolysis (Table S1). Consistent with our study, Abe et al. [41] reported a strong correlation (r = 0.83) between DPPH radical scavenging capacity of fruits consumed by the Brazilian Population and TEA content. However, a weak correlation was found between the TPC and DPPH, as well as vitamin C and DPPH (r~0.50, *p* = 0.01).

### 3.5. Principal Component Analysis of Correlated Variables and Variance among the Trees

PC1 and PC2, as shown in Figures S1 and S2, collectively explained 75% of the total variance in the dataset. The correlation loading indicates that all variables, except TFC, significantly contribute to the PCs. Some fruits picked in Karajarri (K2, K3, and K5) and Yawuru (Y4) play a substantial role in PC1, elucidating 56% of the sample variance (Figure S2). FRAP, TPC, and vitamin C are clustered on the right side of PC1, consistent with Table S1 correlation results. PC2, accounting for 18% of the variance, is notably influenced by FEA and TEA, all highly correlated.

The Bi-plot in Figure S2 reveals that four fruit samples from Yawuru trees (Y2, Y3, Y4, Y5) and five from Karajarri trees (K2, K3, K4, K6, and K9) reside on the right side (regions B and D) of PC1. The rest cluster on the left (A and C) of PC1. The largest negative contribution for PC1 comes from K5, with fruits exhibiting the lowest FRAP, TPC, and vitamin C. Trees in the upper part of regions A and B (Y2, Y3, and Y6) yield fruits with the highest FEA and TEA. Meanwhile, those positioned mid to far right in B and D on PC1 produce fruits rich in antioxidants and vitamin C. Negative PC2 values highlight that sample K10 has high vitamin C compared to Y2, Y6, and Y3. These indicate that vitamin C, TPC, and EA contents are crucial variables for selecting KP trees. Furthermore, fruits with elevated vitamin C, TPC, and EA tend to exhibit superior antioxidant activities in FRAP and DPPH scavenging assays.

### 3.6. Antimicrobial Properties

Trees that gave low and high EA levels were selected from each location (Table 2) for antimicrobial assay. The findings, displayed in Table 4, showed that the maximum zone of inhibition was against MRSA2, *S. aureus,* and *S. putrefaciens*. Interestingly, all the fruit extracts were of weak to moderate activity against *P. aeruginosa* and *A. baumannii*. This is consistent with a previous study on KP, where Gram-negative bacteria were resistant to fruits and leaf extracts [42]. Interestingly, oxytetracycline as a standard antibacterial agent remained inactive against P. aeruginosa.

To explore the KP fruit’s phytochemical potential role in antimicrobial properties, correlation coefficient analysis was conducted and is presented in Table S2. The results showed that EA (R2 = 0.6 to 0.83) and TPC (R2 = 0.57 to 0.76) were significantly correlated (*p* < 0.05) with antibacterial properties viz MRSA2, *S. aureus*, and *S. putrefaciens*. These findings were well supported by several studies [43]. Further, Savic et al. [43] revealed that EA did not have activity against gram negative bacteria (*E. coli* and *P. aeruginosa*), probably explaining the ineffectiveness of KP fruit extracts viz tested Gram-negatives (Table 4). The MIC (Table 4) was determined to be 4 mg/mL; while slightly less than the KP from NT (3 mg/mL), it might be linked to variations in EA and ET contents [10]. However, the Kimberley fruits stand out as the most potent antimicrobial agent compared to persimmon (312.50 mg/mL), guava (1250.0 mg/mL), and sweetsop (1250.00 mg/mL), against *S. aureus* [44], showing intriguing potential in fighting microbial infections.

### 3.7. α-Glucosidase Inhibition

**Figure 1:**
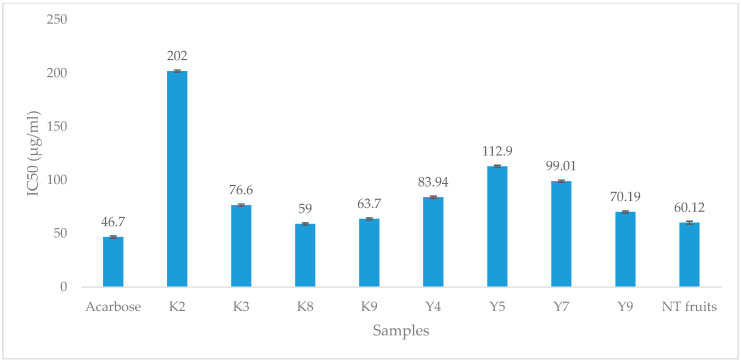
The IC_50_ values of the KP fruits wild harvested from Karajarri (K) and Yawuru (Y), Kimberley region, Western Australia. T: tree, NT: Northern Territory. Values are expressed as mean ± SEM of triplicate measurements.

We examined the inhibition of the α-glucosidase enzyme, which plays a vital role in controlling glucose absorption, especially following meals in individuals diagnosed with diabetes mellitus [45]. Figure 1 shows the α-glucosidase inhibition results. All of the fruit’s extracts suppressed the enzyme activities. Three of the Karajarri samples (trees 3, 8, and 9), two of the Yawuru samples (trees 4 and 9), and NT fruits showed the highest activity. The most potent inhibitors were KP fruits wild harvested from NT and Karajarri. The IC_50_ of fruits picked from the eighth, ninth, and third trees and NT fruits were 59 ± 1.1, 64 ± 1, 76.6 ± 1.0, and 60.1 ± 1.4 µg/mL, respectively. Only one tree, tree 9 (IC_50_ = 70.2 ± 1.0 µg/mL), found in Yawuru, bore fruits that exhibited the level of potency closer to Karajarri. The least potent extracts obtained from Yawuru, displayed in our sample, exhibited the α-glucosidase inhibitory activities comparable with *T. catappa* fruits (115.8 ± 4.3 µg/mL) [46]. There is almost a two-fold difference between the least potent fruits (tree 2, 202 ± 1.0 µg/mL) from Karajarri and the fruits from Yawuru (tree 5, 112 ± 1.1 µg/mL). However, the potency observed among fruits picked from the second tree of Karajarri is even superior to the inhibitory activities of *A. deliciosa* (21.7 ± 1.0 mg/mL), *C. frutescens* (13.19 ± 5.4 mg/mL), and *P. nigrum* (1.36 ± 0.3 mg/mL) [47].

In more recent studies, punicalagin, chebulinic acid, and chebulinic acid, isolated from *T. chebula*, showed significant α-glucosidase inhibitory activities [48,49]. Similar compounds (Table 2) might act individually or along with other compounds to enhance these properties, shown in Figure 1.

## 4. Conclusions

This study, for the first time, assessed the metabolite content and bioactivities of KP fruits wild harvested from Western Australia (WA). The results demonstrated that WA KP fruits represent a unique edible source of glucose, fructose, sucrose, EA, and vitamin C. The fruits also exhibited strong antioxidant activities and activity against microbes responsible for food spoilage and diseases. A promising antidiabetic effect was noted. A Pearson correlation analysis revealed that phenolic compounds and vitamin C played pivotal roles in delineating the FRAP difference among KP fruits harvested from the two sublocations, whereas the DPPH radical scavenging potential was linked to the total EA content. In essence, this study highlights that TPC, EA, ET, and vitamin C are critical variables to be considered in selecting high-quality KP fruits across diverse locations and environments. This study also recommends further study on the Kakadu plum fruit to bring it from the wild to the plate.

## Figures and Tables

**Table 1:** Total water-soluble carbohydrate, glucose, fructose, and sucrose content of Kakadu plum fruit wild harvested in Western Australia and Northern Territory (*n* = 3).

Trees/Region	Percent (%) Dry Weight *		Carbohydrate (mg Glu E/g DW)		Sugar Content ^€^			
					Glucose (g/100 g DW)	Fructose (g/100 g DW)	Sucrose (g/100 g DW)	Total Sugar (g/100 g DW)
	Karajarri	Yawuru	Karajarri	Yawuru				
1	0.1540	0.1703	584.00 ± 0.84 ^d^	449.7 ± 15.8 ^cd^	5.1 ± 0.3	6.2 ± 0.4	5.3 ± 0.1	16.7 ± 0.5
2	0.1470	0.1692	289.5 ± 11.8 ^a^	374.7 ± 11.85 ^b^	7.2 ± 0.4	7.3 ± 0.2	2.7 ± 0.1	17.2 ± 0.2
3	0.1600	0.1390	296.60 ± 8.80 ^a^	350.3 ± 4.2 ^b^	8.0 ± 0.6	7.8 ± 0.5	4.4 ± 0.3	20.2 ± 0.7
4	0.1420	0.1576	396.6 ± 10.10 ^c^	253.4 ± 12.15 ^a^	9.3 ± 0.4	9.1 ± 0.6	3.8 ± 0.2	22.2 ± 0.7
5	0.1502	0.1685	385.3 ± 9.8 ^bc^	284.0 ± 7.02 ^a^	6.2 ± 0.2	7.2 ± 0.3	0.9 ± 0.01	14.3 ± 0.2
6	0.1361	0.1870	364.8 ± 7.7 ^bc^	411.2 ± 13.54 ^bc^	6.7 ± 0.5	8.0 ± 0.1	3.3 ± 0.2	18.0 ± 0.4
7	0.1451	0.1672	389.3 ± 16.0 ^c^	387.7 ± 6.50 ^bc^	5.0 ± 0.3	6.0 ± 0.3	1.6 ± 0.1	12.6 ± 0.3
8	0.1783	0.1684	377.0 ± 6.9 ^bc^	403.4 ± 6.0 ^bc^	6.8 ± 0.1	7.5 ± 0.5	3.1 ± 0.1	17.5 ± 0.3
9	0.1770	0.1795	334.2 ± 8.4 ^ab^	366.4 ± 11.40 ^b^	5.7 ± 0.1	7.2 ± 0.3	0.2 ± 0.01	13.1 ± 0.3
10	0.1684	0.1676	398.1 ± 2.0 ^c^	485.60 ± 5.24 ^d^	6.2 ± 0.3	6.8 ± 0.6	1.1 ± 0.1	14.1 ± 0.5
WA average	0.16 ± 0.01		379.05 ± 18.5		6.6 ± 0.3	7.3 ± 0.4	2.6 ± 0.1	16.6 ± 0.4
NT	0.17 ^α^		253.4 ± 57.0 ^#^		2.0 ± 0.4	3.0 ± 0.4	0.2 ± 0.02	5.2 ± 0.8

Values are expressed as mean ± SEM of triplicate measurements. * Weight of seed excluded, mg Glu E/g DW: - milligram glucose equivalent per gram dry weight, **^€^** determined by randomly selecting 5 trees from Karajarrii and 5 trees from Yawuru, K: - Karajarri country, Y: - Yawuru conservation, WA: Western Australia, NT: - Northern Territory, ^α, #^ data obtained from Konczak et al. [4]. Different letters in the same column show significant differences at *p* < 0.05.

**Table 2:** Ellagic acid and vitamin C contents of Kakadu plum fruit wild harvested in Kimberley, Western Australia (*n* = 3).

Trees	Ellagic Acid				Vitamin C			
	FEA (mg/g DW)		TEA (mg/g DW)		L-AA (mg/g DW)		TVC (mg/g DW)	
	Karajarri	Yawuru	Karajarri	Yawuru	Karajarri	Yawuru	Karajarri	Yawuru
1	20.52 ± 0.50 ^bcd^	16.60 ± 0.75 ^bc^	24.26 ± 0.80 ^a^	30.81 ± 0.45 ^c^	59.63 ± 1.00	47.10 ± 0.30	61.44 ± 0.63	50.32 ± 1.51
2	24.60 ± 0.32 ^ef^	31.30 ± 0.22 ^f^	29.42 ± 0.50 ^ab^	49.22 ± 1.30 ^h^	126.43 ± 0.51	54.2 ± 0.47	131.50 ± 0.20	57.12 ± 0.64
3	30.40 ± 0.40 ^g^	34.40 ± 0.43 ^g^	42.74 ± 0.31 ^de^	47.40 ± 0.60 ^gh^	110.70 ± 1.23	66.8 ± 0.22	115.00 ± 1.40	69.5 ± 0.30
4	22.70 ± 0.07 ^cde^	30.01 ± 0.60 ^f^	33.41 ± 0.54 ^bc^	43.30 ± 0.81 ^fg^	94.83 ± 0.43	118.63 ± 2.75	98.00 ± 1.00	122.33 ± 3.25
5	15.00 ± 0.40 ^a^	24.75 ± 0.25 ^d^	23.30 ± 1.4 ^a^	36.10 ± 0.60 ^de^	24.51 ± 0.01	87.65 ± 0.68	25.20 ± 0.16	90.82 ± 1.10
6	23.10 ± 0.50 ^de^	31.70 ± 0.34 ^f^	41.00 ± 0.75 ^de^	42.70 ± 2.30 ^fg^	111.51 ± 0.42	40.95 ± 0.10	114.20 ± 0.74	42.47 ± 0.27
7	20.00 ± 0.24 ^bc^	12.28 ± 0.60 ^a^	33.32 ± 0.80 ^bc^	19.00 ± 0.40 ^a^	55.60 ± 0.44	61.22 ± 0.31	57.77 ± 0.74	63.70 ± 0.25
8	18.64 ± 0.33 ^b^	27.24 ± 0.42 ^e^	40.53 ± 3.61 ^cd^	40.00 ± 0.12 ^ef^	95.00 ± 0.80	57.05 ± 0.30	97.40 ± 0.60	58.27 ± 0.40
9	26.64 ± 0.30 ^f^	14.80 ± 0.43 ^b^	44.62 ± 1.00 ^e^	31.63 ± 0.80 ^cd^	77.30 ± 1.22	53.56 ± 0.22	81.00 ± 1.20	54.03 ± 0.13
10	19.53 ± 1.82 ^bcd^	17.60 ± 0.10 ^c^	28.52 ± 0.40 ^ab^	26.00 ± 0.35 ^b^	75.40 ± 1.43	53.20 ± 2.80	80.71 ± 3.60	54.00 ± 3.0
Average	22.2 ± 0.8	24.1 ± 2.5	34.0 ± 1.4	36.6 ± 3.1	83.1 ± 5.4	64.0 ± 7.3	86.2 ± 5.6	66.1 ± 7.5

Values are expressed as mean ± SEM of triplicate measurements. Different letters in the same column show significant difference at *p* < 0.05. FEA: free ellagic acid, TEA: total ellagic acid, L-AA: L-ascorbic acid, TVC: total vitamin C.

**Table 3:** Total phenolic (TPC), total flavonoid (TFC), ferric reducing antioxidant power of plasma (FRAP) and 2,2-diphenyl-1-picrylhydrazyl (DPPH) radical scavenging power of Kakadu plum fruit wild harvested in Kimberley, Western Australia (*n* = 3).

Trees	TPC (mg GAE/g DW)		TFC (mg QE/g DW)		FRAP (µmole Fe^2+^ E/g DW)		DPPH (µmole TE/g DW)	
	Karajarri	Yawuru	Karajarri	Yawuru	Karajarri	Yawuru	Karajarri	Yawuru
1	112.70 ± 2.70 ^bc^	110.72 ± 2.70 ^ab^	4.00 ± 0.06 ^cd^	5.30 ± 0.04 ^d^	1422.72 ± 44.90 ^b^	1235.75 ± 23.70 ^a^	1204.50 ± 7.23 ^a^	1157.00 ± 8.44 ^ab^
2	184.71 ± 4.80 ^e^	133.22 ± 2.00 ^cd^	4.10 ± 0.05 ^de^	5.62 ± 0.08 ^e^	2315.55 ± 55.12 ^d^	1565.20 ± 32.33 ^bc^	1274.13 ± 5.41 ^ab^	1288.23 ± 16.00 ^bc^
3	190.50 ± 3.20 ^e^	124.00 ± 2.00 ^bc^	3.53 ± 0.05 ^ab^	3.13 ± 0.05 ^a^	2266.12 ± 53.00 ^d^	1530.23 ± 24.00 ^b^	1381.00 ± 14.40 ^bc^	1271.27 ± 47.41 ^bc^
4	165.60 ± 3.00 ^d^	195.40 ± 3.30 ^e^	3.74 ± 0.05 ^bc^	5.04 ± 0.08 ^d^	1979.26 ± 43.00 ^c^	2350.20 ± 60.25 ^d^	1313.30 ± 19.22 ^abc^	1390.50 ± 18.10 ^c^
5	77.74 ± 2.00 ^a^	147.50 ± 4.00 ^d^	4.20 ± 0.05 ^de^	5.10 ± 0.05 ^d^	1011.20 ± 24.10 ^a^	1711.92 ± 53.33 ^c^	1229.93 ± 30.13 ^a^	1314.40 ± 29.60 ^bc^
6	160.70 ± 3.43 ^d^	106.55 ± 3.34 ^a^	3.80 ± 0.07 ^bc^	5.72 ± 0.06 ^e^	1943.82 ± 42.60 ^c^	1288.05 ± 33.00 ^a^	1431.87 ± 30.23 ^c^	1300.30 ± 32.82 ^bc^
7	104.65 ± 3.45 ^b^	111.70 ± 3.70 ^ab^	4.6 ± 0.06 ^f^	6.54 ± 0.09 ^f^	1350.10 ± 45.00 ^b^	1257.11 ± 54.01 ^a^	1303.56 ± 36.20 ^abc^	1005.47 ± 28.00 ^a^
8	120.54 ± 3.20 ^bc^	99.52 ± 7.70 ^a^	4.34 ± 0.05 ^ef^	3.90 ± 0.04 ^b^	1542.46 ± 31.70 ^b^	1250.50 ± 37.00 ^a^	1314.80 ± 44.00 ^abc^	1269.40 ± 46.54 ^bc^
9	154.20 ± 4.00 ^d^	113.10 ± 2.00 ^ab^	3.60 ± 0.05 ^b^	3.32 ± 0.06 ^a^	1803.21 ± 34.12 ^c^	1353.20 ± 32.00 ^a^	1410.00 ± 39.03 ^c^	1212.81 ± 24.66 ^bc^
10	126.60 ± 5.00 ^c^	103.10 ± 2.30 ^a^	3.30 ± 0.08 ^a^	4.44 ± 0.03 ^c^	1488.20 ± 33.00 ^b^	1255.32 ± 15.05 ^a^	1223.11 ± 28.30 ^a^	1208.35 ± 33.44 ^bc^
Average	140.0 ± 6.6	124.5 ± 9.2	3.9 ± 0.07	4.8 ± 0.3	1714.4 ± 74.4	1476.7 ± 110.6	1311.2 ± 17.2	1242.8 ± 33.2

Values are expressed as mean ± SEM of triplicate measurements. K: Karajarri, Y: Yawuru; mg GAE/g DW: milligram gallic acid equivalent per gram dry weight; mg QE/g DW: milligram quercetin equivalent per gram dry weight; µmole Fe^2+^ E/g DW: micromole ferrous equivalent per gram dry weight; µmole TE/g DW: micromole Trolox equivalent per gram dry weight. Different letters in the same column show significant difference at *p* < 0.05.

**Table 4:** Antimicrobial activities of selected Kakadu plum fruit (picked from Karajarri (K) and Yawuru (Y) trees) extracts (40 mg/mL) against spoilage and pathogenic microorganisms (*n* = 3).

Trees/Std	Zone of Inhibition Measured in mm					MIC (mg/mL)		
	MRSA2	SA	PA	AB	S. put	SA	MRSA2	S. put
4Y	13.8 ± 0.3 ^c^	14.4 ± 0.6 ^c^	6.1 ± 0.7 ^a^	6.0 ± 0.9 ^a^	10.0 ± 0.2 ^b^	4	4	4
5Y	12.2 ± 0.8 ^abc^	12.0 ± 0.1 ^b^	7.0 ± 0.5 ^a^	8.0 ± 0.5 ^a^	10.0 ± 0.5 ^b^	4	4	4
7Y	10.2 ± 0.9 ^a^	10.0 ± 0.7 ^a^	6.0 ± 0.1 ^a^	6.6 ± 0.4 ^a^	7.8 ± 0.2 ^a^			
9Y	13.0 ± 1.0 ^bc^	10.5 ± 0.8 ^ab^	6.5 ± 0.5 ^a^	7.0 ± 1.0 ^a^	10.0 ± 0.3 ^b^	4	4	4
2K	12.7 ± 0.6 ^AB^	13.3 ± 0.5 ^C^	8.0 ± 0.1 ^B^	8.3 ± 0.6 ^B^	9.5 ± 0.6 ^BC^	4	4	4
3K	14.1 ± 0.5 ^B^	14.7 ± 0.3 ^CD^	6.0 ± 0.1 ^A^	8.2 ± 0.7 ^B^	13.4 ± 0.9 ^D^	4	4	4
8K	11.1 ± 0.4 ^A^	8.4 ± 0.3 ^A^	6.0 ± 0.7 ^A^	5.1 ± 0.6 ^A^	6.8 ± 0.3 ^A^			
9K	13.6 ± 0.5 ^B^	15.1 ± 0.8 ^D^	6.0 ± 0.1 ^A^	8.5 ± 0.3 ^B^	11.0 ± 0.8 ^C^	4	4	4
Oxy (0.06 mg/mL)	22.1 ± 1.0	28.8 ± 0.4	-	8.7 ± 0.3	32.6 ± 0.5			

Different small letters in the same column showed a significant difference among fruits picked from Yawuru. Different capital letters in the same column showed significant difference among fruits picked from Karajarri. Oxy: Oxytetracycline, K: Karajarri, Y: Yawuru, MRSA2: *Methicillin resistant staphylococcus aureus*, SA: *Staphylococcus aureus*, PA: *Pseudomonas aeruginosa*, AB: *Acinetobacter baumannii*, S. put: *Shewanella putrefaciens*.

## Data Availability

The original contributions presented in the study are included in the article/Supplementary Material, further inquiries can be directed to the corresponding authors.

## Supplementary Materials

The following supporting information can be downloaded at: https://www.mdpi.com/article/10.3390/foods13182888/s1, Figure S1: PCA loadings; Figure S2: PCA bi-plot; Table S1: Correlation between metabolites and antioxidant activities; Table S2: Correlation between phytochemicals and antimicrobial activities.
