# Supplementary material for: Nutritional and Functional Properties of Terminalia ferdinandiana Fruits Wild Harvested from Western Australia
Source: Foods. 2024 Sep 12;13(18):2888. doi: 10.3390/foods13182888 (PMC11431513; doi:10.3390/foods13182888)
Supplement: Supplementary file 1 [file foods-13-02888-s001.zip › foods-3173231-supplementary.pdf]

Supplementary material

Table S1. Pearson correlation between TPC (mg GAE/g DW), ellagic acid (mg/g DW), vitamin C (mg/g DW) and antioxidant activities (DPPH and FRAP Assays) of Kakadu plum fruit wild harvested in Karajarri and Yawuru, Western Australia.

**Variables**	**Karajarri Tree**		**Yawuru Tree**	
	FRAP	DPPH	FRAP	DPPH
**TPC**	0.99	0.49	0.97	0.50
**FEA**	0.84	0.53	0.50	0.69
**TEA**	0.53	0.82	0.45	0.73
**L-AA**	0.92	0.40	0.91	0.42
**TVC**	0.92	0.41	0.91	0.42

TPC: - Total phenolic contents, FEA: - free ellagic acid contents, TEA: - total ellagic acid contents, L-AA: - L-ascorbic acid, TVC: - total vitamin C. The correlation is significant at *p=0.01*.

Table S2. Pearson correlation coefficients between the phytochemical contents and the antimicrobial properties of the analysed fruit extracts.

		**Yawuru Correlation Matrix**			**Karajarri Correlation Matrix**		
	Variables	*MRSA2*	*SA*	*S. put*	*MRSA2*	*SA*	*S. put*
**Correlation**	FEA	0.600**	0.816**	0.507*	0.825**	0.820**	0.750**
	TEA	0.779**	0.748**	0.595**	0.467	0.317	-0.012
	TPC	0.570*	0.755**	0.035	0.713**	0.748**	0.756**
	TFC	-0.437	-0.094	-0.714**	-0.296	-0.458	-0.539*
	LAA	0.538*	0.785**	0.017	0.261	0.149	0.212
	TVC	0.524*	0.775**	-0.001	0.276	0.156	0.235

**Correlation is significant at the $p < 0.01$ level, * Correlation is significant at the $p < 0.05$ level, FEA: - free ellagic acid, TEA: -total ellagic acid, TPC: -total phenolic contents, TFC: -total flavonoid contents, L-AA: -L-ascorbic acid, TVC: -total vitamin C, *MRSA2*: - *methicillin resistant staphylococcus aureus*, *SA*: - *Staphylococcus aureus*, *S. put: - Shewanella putrefaciens*

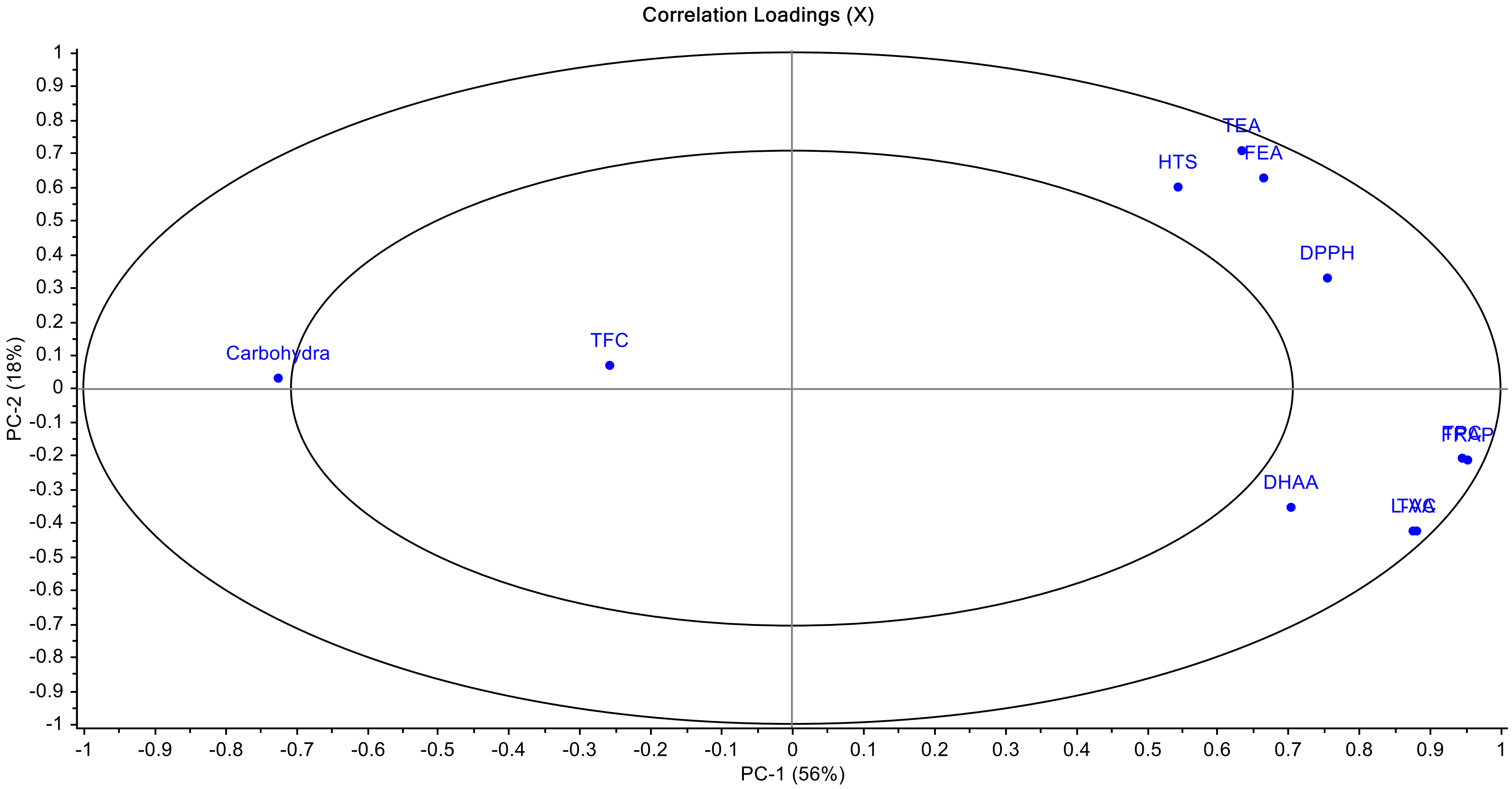

Figure S1. Principal component analysis (PCA) correlation loadings of Karajarri (K) and Yawuru (Y) trees.

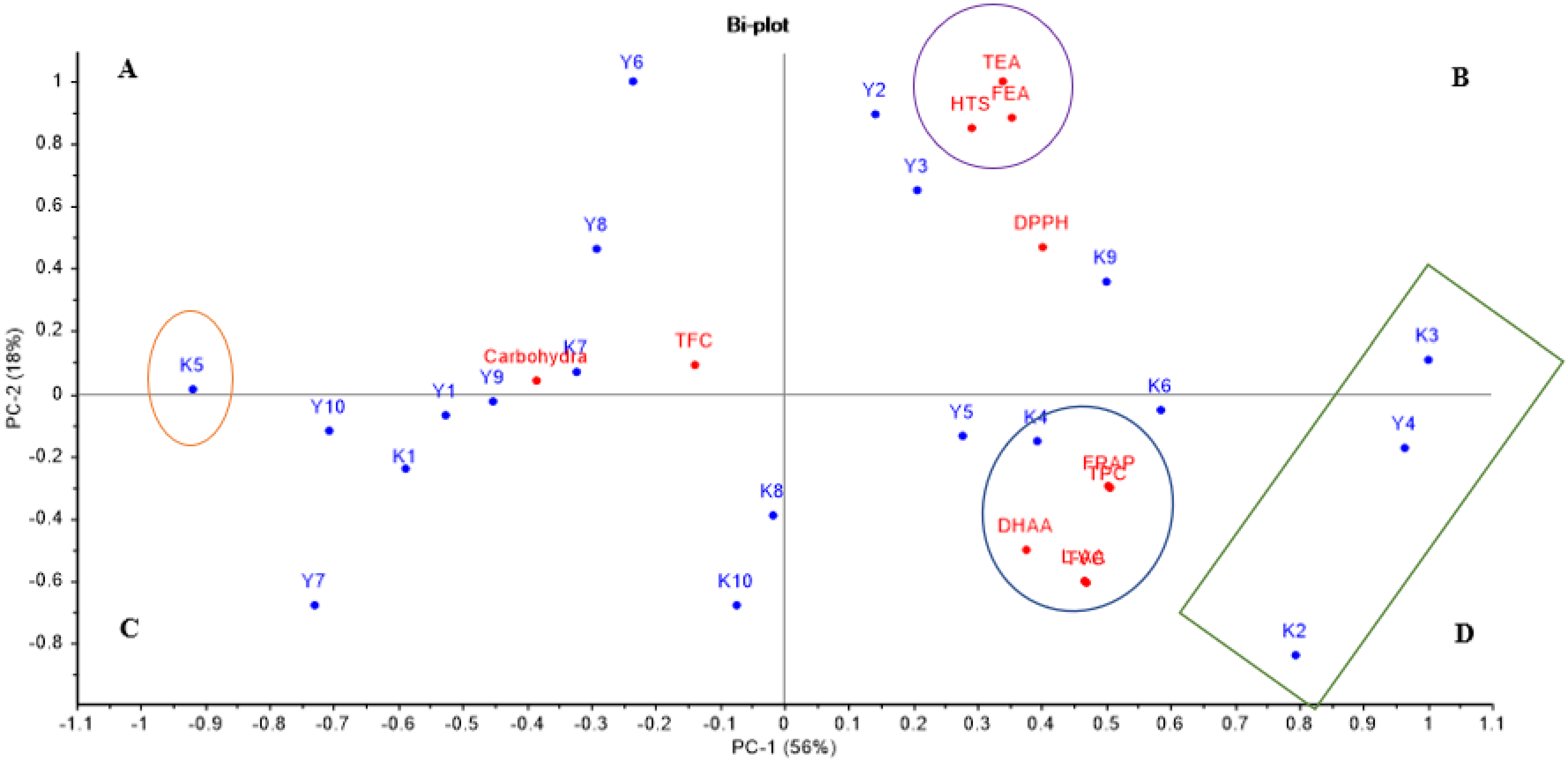

Figure S2. Principal component analysis (PCA) Bi-plot of Karajarri (K) and Yawuru (Y) trees. TEA-total ellagic acid, FEA-free ellagic acid, HTS-hydrolysable tannins, FRAP-Ferric reducing power of plasma, TPC-total phenolic content, TVC-total vitamin C, L-AA-L-ascorbic acid, TVC-total vitamin C, TFC-total flavonoid content, Carbohydra-total carbohydrate contents.
